# Expression of Emotional Arousal in Two Different Piglet Call Types

**DOI:** 10.1371/journal.pone.0135414

**Published:** 2015-08-14

**Authors:** Pavel Linhart, Victoria F. Ratcliffe, David Reby, Marek Špinka

**Affiliations:** 1 Department of Ethology, Institute of Animal Science, Prague, Czechia; 2 School of Psychology, University of Sussex, Brighton, United Kingdom; Texas Christian University, UNITED STATES

## Abstract

Humans as well as many animal species reveal their emotional state in their voice. Vocal features show strikingly similar correlation patterns with emotional states across mammalian species, suggesting that the vocal expression of emotion follows highly conserved signalling rules. To fully understand the principles of emotional signalling in mammals it is, however, necessary to also account for any inconsistencies in the way that they are acoustically encoded. Here we investigate whether the expression of emotions differs between call types produced by the same species. We compare the acoustic structure of two common piglet calls—the scream (a distress call) and the grunt (a contact call)—across three levels of arousal in a negative situation. We find that while the central frequency of calls increases with arousal in both call types, the amplitude and tonal quality (harmonic-to-noise ratio) show contrasting patterns: as arousal increased, the intensity also increased in screams, but not in grunts, while the harmonicity increased in screams but decreased in grunts. Our results suggest that the expression of arousal depends on the function and acoustic specificity of the call type. The fact that more vocal features varied with arousal in scream calls than in grunts is consistent with the idea that distress calls have evolved to convey information about emotional arousal.

## Introduction

Vocalizations have long been considered as a window into emotional states in both humans and other animals [1]. Indeed, emotion-specific calls can be triggered by the stimulation of basic emotional brain systems [2], while vocalizations alone can induce appropriate behavioural and physiological responses [3]. Because of their close association with emotional expression, vocalizations and their specific parameters have received significant scientific attention as possible indicators of emotional states in animals [4,5]. Recently, a dimensional approach has been advocated in characterising animal emotional states [6]. The most common model contains two core affect dimensions: arousal, which quantifies the intensity of the response, and valence, which quantifies the aversiveness of the situation—ranging from a very positive (pleasurable) to a very negative (aversive) emotional state. The dimensional approach provides a framework in which the signalling of emotional states can be easily compared across different species.

When animals are exposed to different emotionally loaded situations, the acoustic features of their calls vary with the assumed emotional valence and/or emotional arousal [4]. Particularly, the pattern of changes of certain vocal features along the arousal gradation is surprisingly uniform among species (for example both pitch and amplitude increase with arousal), suggesting common underlying mechanisms [4, 5]. This remarkable consistency is likely due to the fact that the principles of vocal production [7,8] and perception [9,10] are conservative in mammalian species. Interestingly however, there are some call parameters, such as call duration or tonality, that do not show such a consistency across animal species [4].

One aspect of emotional signalling which has not yet been considered is that emotional states may be encoded differently across call types within the repertoire of the same species. While call types with qualitatively different acoustic structures or functions, such as alarm calls (e.g. [11]), contact calls (e.g. [12]), or non-situation-specific calls like dog barks [13] or pig grunts (e.g. [14]) have all been investigated (reviewed by [4,5]), distress calls have received disproportionately greater scrutiny (e.g. [15,16,17]), perhaps because animals vocalize more readily and conspicuously when they experience stress. However, because call types are defined by overall differences in their acoustic structure, assumed to reflect specific functions [18], variation in emotional state may be encoded in different ways across call types.

Emotions induce changes in the autonomic nervous system. Changes in emotional arousal trigger modifications in physiological processes such as respiration, muscle tension and salivation [19,20]. Because these processes are directly involved in vocal production, acoustic parameters that are controlled by these processes should vary consistently with the emotional state of the signaller independently from the particular call type produced. For instance, emotionally driven variation in respiratory air flow may similarly modulate the amplitude of vocalisations across call types. Thus, any call type might reflect the emotional state of the animal independently of its function or specific structure [14]. However, few studies have reported how different call types may encode the same emotional state in mammals [14,21].

**Fig 1:**
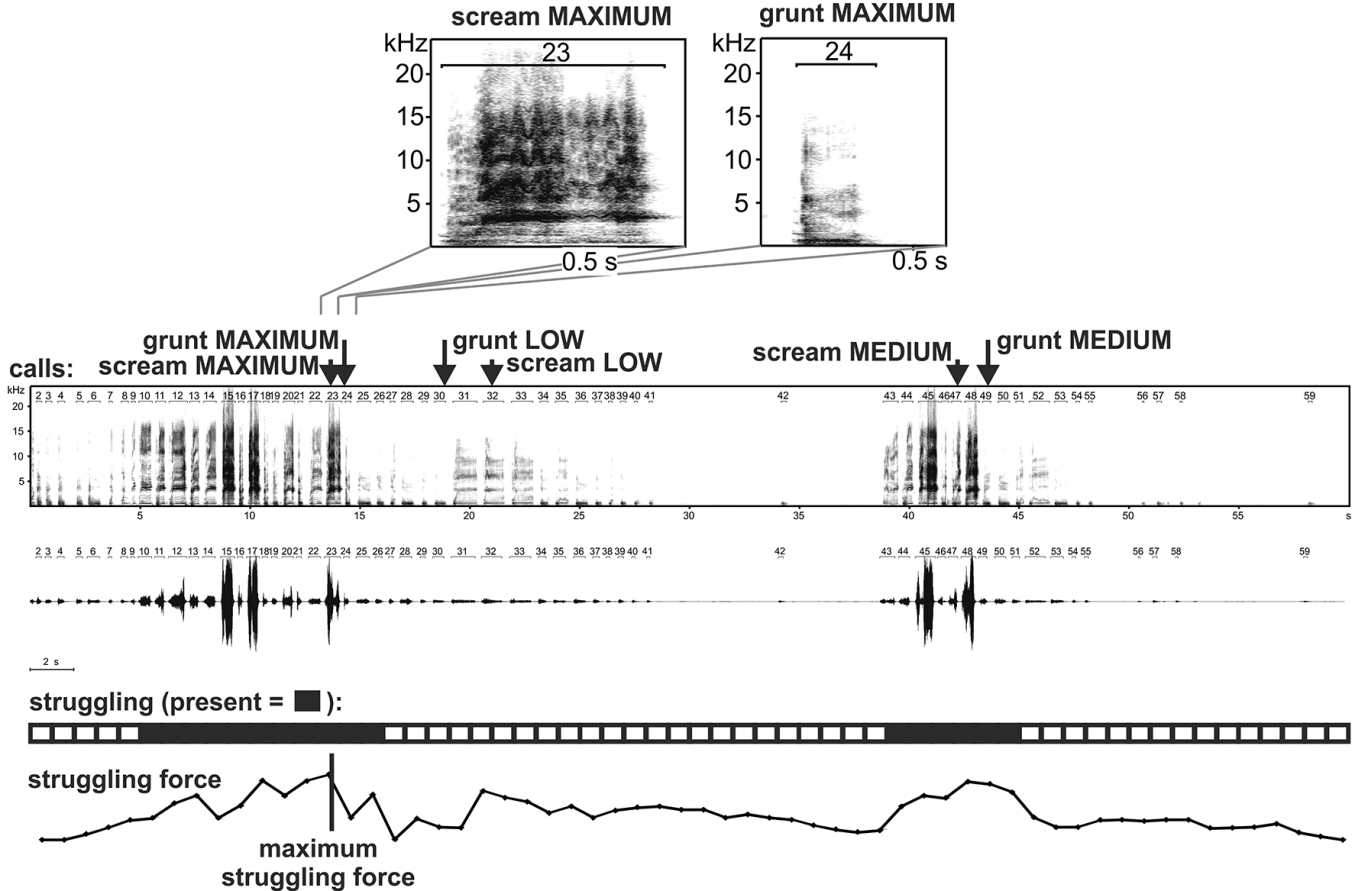
Typical scream and grunt calls and the course of the backtest session, depicting the calling activity (spectrogram and oscillogram), struggling presence, and struggling force over 60s of the backtest on a second by second basis.

We tested the hypothesis that acoustic parameters within two functionally and structurally different call types would change in a similar way with increasing emotional arousal in domestic piglet vocalisations. Piglets emit two basic call types (Fig 1). High frequency calls (screams and squeals) are mainly given in situations of urgent threat. In contrast low frequency calls (grunts) are not situation-specific as they are produced across a wide range of non-emergency social situations as well as urgent distress situations [22]. The signalling function of grunts in distress contexts remains unclear, but as social calls it is possible that they could facilitate individual recognition. Although transitional vocalisations are sometimes produced, screams and grunts represent well separated call types [22,23]. In addition to their functional differences, the production of both calls also differs: grunts are emitted nasally while screams are emitted orally, though both are assumed to originate from the vocal folds [24]. The structure of scream calls has already been shown to reflect arousal in piglets (e.g. [16]), while potential associations between grunt call structure and the emotional state of the signaller remain largely unexplored (but see [14,16]).

In the current study, we directly compare the acoustic structure of piglet screams and grunts emitted in the same emotional and physiological contexts. To control for potential differences in emotional state between individuals, we compare scream and grunt calls that were emitted in immediate succession across three broad levels of arousal, ensuring that the two vocalisations were produced when the piglet was in an equivalent physiological state. As the purpose of this study is to demonstrate general principles, rather than to determine specific indicators of particular emotional states, we focus on a small number of commonly investigated acoustic parameters: duration, amplitude, central frequency and harmonic-to-noise ratio. In accordance with previous research, we expect that both grunts and screams should be longer, more intense, harsher (lower harmonic-to-noise ratio) and have more energy in higher frequencies with increasing arousal.

## Methods

### Ethics statement

The experiment was approved by the Institutional Animal Care and Use Committee of the Institute of Animal Science and the Czech Central Committee for Protection of Animals, Ministry of Agriculture (decision MZe 1244). This study was a part of larger project investigating the consistency of arousal signalling across different contexts and situations. For this reason, we recorded piglets in a number of positive and negative natural and experimental situations with different levels of arousal. The backtest situation (commonly used in pigs to study individual coping styles [25,26,27]) corresponds to a mildly stressful situation caused by the separation of the piglet from its litter and being placed in an unnatural position. The stress induced by the backtest is considered mild and is not prolonged (the whole procedure, including handling, lasts about 3 minutes). All of the piglets were healthy when they were returned to their home pen immediately after the test. Piglets were carefully and gently handled and transported in a straw-bedded crate to reduce stress. No pain relievers were necessary.

### Animals

Piglets (Large White × Landrace) were born and kept at the experimental farm of the Institute of Animal Science, Prague, Czechia. Sows and piglets were housed in 2.3m x 2m straw-bedded, concrete floor pens equipped with a ‘walk around’ ellipsoid farrowing crate (2.3 m x1.4 m). Sows were fed a standard lactation diet twice a day. Water was available ad libitum separately for the sow and for the piglets. Piglets were weighed and individually marked at day 1 postpartum (body marker). We aimed to test eight piglets per litter. Overall, 12 litters were subjected to backtest. In 3 litters, we could only test 6, 6, and 4 piglets per litter due to small litter size or very low weight (< 1.00 kg) leading to 88 piglets with intermediate weights (mean +- sd = 1.84 kg +- 0.44 kg; range: 1.04 kg–2.80 kg) being subjected to backtest. The number of male and female piglets was balanced (44 males / 44 females).

Each litter was tested within a single pause between nursing episodes starting 5 minutes after nursing ended. Piglets were tested in a randomized order. Each piglet was gently removed from the litter and transported (c.a. 30 s) in a straw-bedded basket to a separate testing room where the backtest was performed. In general, piglets remained calm and silent during pen removal and transportation. The whole procedure took about 3 minutes.

### Backtest

We used the backtest as a model situation, which is commonly used in pigs to study individual coping styles. For the purpose of this study, we were not interested in coping styles of piglets but instead focused on behaviour of the piglets during the backtest. We adapted the procedure from Hessing et al. [27] and Ruis et al. [26]. The piglet was gently put on its back on a digital scale and kept in this position for 60 s by placing one hand over the piglet’s thorax and holding its right foreleg with the other hand. The hind legs remained free to move. The smallest force possible was used to keep the piglet in the supine position to ensure that the weight readings on the scale matched the struggling force of the piglet as closely as possible. One person (PL) performed all of the testing. Although the scale readings are only approximations of the struggling force, they allowed us to unambiguously differentiate periods of more versus less intense struggling. We recorded the number, latency and duration of struggling periods. One struggle was defined as a continuous series of wriggles. Subsequent struggles had to be separated at least by 1 s of rest to be treated as two separate struggles. The whole procedure was videotaped. We noted whether the piglet was struggling or not on a second-by-second basis. We also identified the moment when the piglet was struggling with the maximum intensity (as the absolute maximum weight on the scale).

### Three levels of arousal

Previous studies have used different approaches to induce and assess emotional arousal in animals, including, for instance, presenting more and less dangerous predators, avoidance / preference for the situation, administration of psychoactive drugs, as well as using the intensity of the behavioural and physiological response to situation or stimulus [4,5]. In the present study, we use the struggling of piglets as a behavioural indicator of their emotional arousal. The backtest represents a negatively-valenced, stressful situation leading to an increase in cortisol levels in piglets [28]. Piglet's struggling to escape can be viewed as a behaviour activated by the punishment avoidance system [6]. The responses of piglets to the backtest vary from tonic immobility, lying still but alerted, to fierce struggling. These levels correspond well with the description of arousal by Russel [29]: “The vertical dimension, arousal, ranges from sleep, then drowsiness, through various stages of alertness to frenetic excitement. The feeling is one’s sense of mobilization and energy.” Although we use only behavioural indicators of arousal, it is very likely that the frequently used physiological indicators of arousal such as heart rate and respiration rate (see e.g. [30]) would reflect struggling intensity as well. We therefore consider struggling as a good indicator of arousal in the backtest situation.

For each piglet, we identified three broad arousal levels during the backtest based on qualitative and quantitative behavioural indicators. The backtest situation is assumed to be experienced as negative and unpleasant, yet not painful, by piglets. The behaviour of piglets during the test varied markedly from lying still (= no or little muscle activation) to intense struggling, indicating a progressive activation of the punishment avoidance system [6]. We identified three distinct arousal levels: 1) Low arousal level (LOW): the piglet lies still on the scale without struggling, the piglet resists the situation passively; 2) medium arousal level (MEDIUM): the piglet shows active resistance and struggles to escape with the low to very high resistance force (we took the weight necessary to keep piglet in a supine position as a proxy for the resistance intensity); 3) maximum situation arousal level (MAXIMUM): the piglet shows the most intensive active resistance whilst struggling (maximum weight necessary to keep piglet in a supine position during the backtest).

### Audio recording and analyses

A directional microphone (Sennheiser ME66, flat response 40–20000 Hz, maximum sound pressure level = 128 dB SPL) equipped with a foam windshield was placed 1.5m above the piglet during the trial. Vocalizations were recorded on a digital solid state recorder Marantz PMD671 into a 24-bit, 48 000 Hz, mono, PCM (wav) format. The recording volume gain was kept constant over the recordings. To measure the sound pressure level, the recordings were calibrated with a reference beep sound whose sound level was measured at the microphone with also-Tech SLM-1352A Sound level meter (fast, A-weighting). Piglets were able to move their heads freely, therefore the head was not always directly pointing in the direction of the microphone, which could have affected the measurements of the sound pressure level. Nevertheless, head movements occurred in all stages of test and hence this variation was unlikely to cause any systematic bias in our results. Recordings were analysed in Avisoft SAS Lab Pro [31]. All calls were identified automatically in the first stage (-15 to -20dB thresholds for call onset and offset depending on the recording). Annotated recordings were then reviewed manually and any misclassifications were corrected (c. a. 1–5%; non-detected calls, partly detected calls–incorrect onsets or offsets, merged calls, etc.).

We selected 6 calls per piglet: 3 scream calls (high frequency calls) and 3 grunt calls (low frequency calls), with one call per arousal level for each call type (Fig 1). We used the peak frequency as a criterion to classify the call as a grunt or a scream in accordance with previous studies [23]. Because the distribution of peak frequency values was clearly bi-modal, calls with a peak frequency above 1000 Hz were classified as screams, whilst the remaining calls were classified as grunts. Scream calls were always selected first for each arousal level. The MAXIMUM scream call was the call emitted in the time of the most intensive struggling resistance +/- one call. The MAXIMUM grunt call was the call immediately neighbouring the MAXIMUM scream or a grunt call produced a maximum of 1s from onset or offset of the scream. Because the MEDIUM arousal level lasted a considerable amount of time and there were typically many MEDIUM scream calls within a trial, we selected one scream at random and paired it with a MEDIUM grunt within 2 s from the MEDIUM scream call. The same selection approach was used in the case of the LOW scream and grunt calls. As a result of this selection process we obtained three pairs of scream and grunt calls per piglet. We did not analyse more than one call from each arousal level as the behaviour of the pigs varied rapidly during the test, for example, the intensity of struggling changed very quickly in the MEDIUM arousal level, whilst calls in LOW arousal level were influenced by their temporal proximity to struggling bouts. Therefore, comparisons based on averages would not suit the purpose of our study. Within each scream/grunt pair we could be sure that the two calls were emitted in a very similar physiological (heart rate, respiration rate, muscle tension, etc.) and emotional / motivational state without the need to measure the physiological and emotional state of the piglets directly. Finally, we had a full set of 6 calls for 30 piglets, providing 180 analyzed calls in total for the main analysis. For the rest of the piglets we were not able to get a complete set of grunts and screams from each arousal level.

We decided to focus our analyses on 4 acoustic parameters of the calls: duration (s); root-mean-square sound intensity (dB SPL); central *frequency (Hz; frequency dividing the average spectrum of the call in two parts of equal energy; 50% quartile frequency in Avisoft SASLab—used as the spectrum distribution characteristic), and tonality (dB, harmonic-to-noise ratio). These four parameters comprise some of the most studied vocal parameters and have previously been linked to specific physiological changes [4]. We did not evaluate the fundamental frequency (F0) because it was problematic to obtain the F0 for a large amount of the calls (because they were too noisy and/or short) and we wanted to focus only on those parameters that would be easily comparable between the scream and grunt call types.

The duration, root-mean-square amplitude and central frequency were measured automatically in the detected calls with the ‘Automatic parameter measurement’ tool in Avisoft SASLab. The spectrogram settings were: FFT length = 1024, window length = 0.046 s, window type = Hamming. The sound intensity was calibrated with a beep of known intensity (see above) at the beginning of the recording by using the function ‘Calibration / SPL with reference sound’ in Avisoft SASLab. The harmonic-to-noise ratio was measured in PRAAT [32] using the ‘To Harmonicity (cc)’ function with the standard settings (Time step: 0.01 s; Minimum pitch: 75 Hz; Silence threshold: 0.1; Periods per window: 1.0). This function computes acoustic periodicity on the basis of a cross-correlation method independently of whether a pitch can be determined [33].

Besides the analyses of acoustic call structure, we were also interested in how piglets vocalize when they were struggling versus when they were still. Because the duration of each behavioural response differed per piglet, we computed the calling rates (grunts/s; screams/s) in each stage (still / struggling) separately.

### Statistical analyses

Statistical analyses were carried out in R. Wilcoxon signed-rank tests were used to determine whether calling rates increased between the struggling and still stages of the backtest. For the main analysis we used calls from 30 piglets for whom we had complete sample of calls (grunts and screams from all three levels of arousal, see also ‘Audio recording and analyses’). We used linear mixed-effect models (‘lme’ function from ‘nlme’ package) to assess the effect of the arousal level on each of the vocal parameters separately (arousal level as fixed factor with three levels: LOW, MEDIUM, and MAXIMUM). The central frequency and duration were first log transformed due to non-normality. We also included an interaction term between call type and arousal level to establish whether any of the acoustic parameters respond differently to changes in arousal depending on the call type. We also added the fixed effects of testing order, piglet sex, and piglet weight to control for their potential influence on the call structure. The test order was included as a covariate because it provided an indication of time after milk intake (we started testing each litter 5 minutes after nursing and the testing of each piglet took about 3 minutes) which could affect the emotional state of piglets [15]. Litter and piglet identity were included as random factors (piglet nested within litter). We used a backwards stepwise method to remove non-significant fixed variables from the models until only significant effects remained. Testing order, piglet sex, and piglet weight were never significant, therefore we report only the models including the fixed effects of arousal level and call type. We checked that the distribution of residuals was approximately normal and did not systematically vary across the fitted values (with constant variance and no trends). There were minor issues with the residual variance in the case of central frequency, suggesting larger variance in the scream calls than in grunt calls. We therefore used the ‘weights’ argument of the ‘lme’ function that enables the heteroscedasticity of the within-error group to be modelled (i.e. allows different variances for each factor level) [34]. We used the ‘ghlt’ function from ‘multcomp’ to perform post-hoc tests within the final linear mixed models.

## Results

### Behaviour of the piglets during the backtest

The behaviour of the piglets is summarized in Table 1. Piglets started to struggle from the start of the backtest (latency to struggle was 4.3 s on average), performed around 3 bouts of struggling during the test (2.7 bouts of struggling on average), and struggled for almost half of the time (25.0 s on average). Piglets also vocalized intensively. On average, piglets emitted mean ± SD = 50.3 ± 21.6 calls during the backtest; taking each call type separately, there were slightly more grunts (29.8 on average) than screams, but the number of screams was also high (20.5 on average).

**Table 1:** Descriptive statistics for piglet behaviour during the backtest.

	all piglets (n = 88)		piglets in analysis (n = 30)	
	mean	sd	mean	sd
struggles (n)	2.71	1.15	2.9	1.12
struggling duration (s)	24.88	12.58	23.07	11.7
latency to struggle (s)	4.3	9.2	3.1	4.3
piglet weight (g)	1830	450	1870	430
maximum resistance force (maximum weight / piglet weight)	1.46	0.16	1.47	0.18
vocalizations (n)	50.3	21.6	56.7	20.1
screams (n)	20.5	18.6	21.9	16.8
grunts (n)	29.8	15.1	34.8	12.9
screams / s—relaxed	0.10	0.17	0.09	0.12
screams / s–struggling	0.67	0.43	0.76	0.32
grunts / s–relaxed	0.35	0.29	0.51	0.32
grunts / s struggling	0.81	0.61	0.78	0.45

The calling rate was higher when the piglets were struggling for both screams (Wilcoxon signed rank test: N = 87, Z = -8.03, r = -0.61, p < 0.001) and grunts (Wilcoxon signed rank test: N = 88, Z = -4.69, r = -0.35, p < 0.001).

### Arousal and call parameters

**Fig 2:**
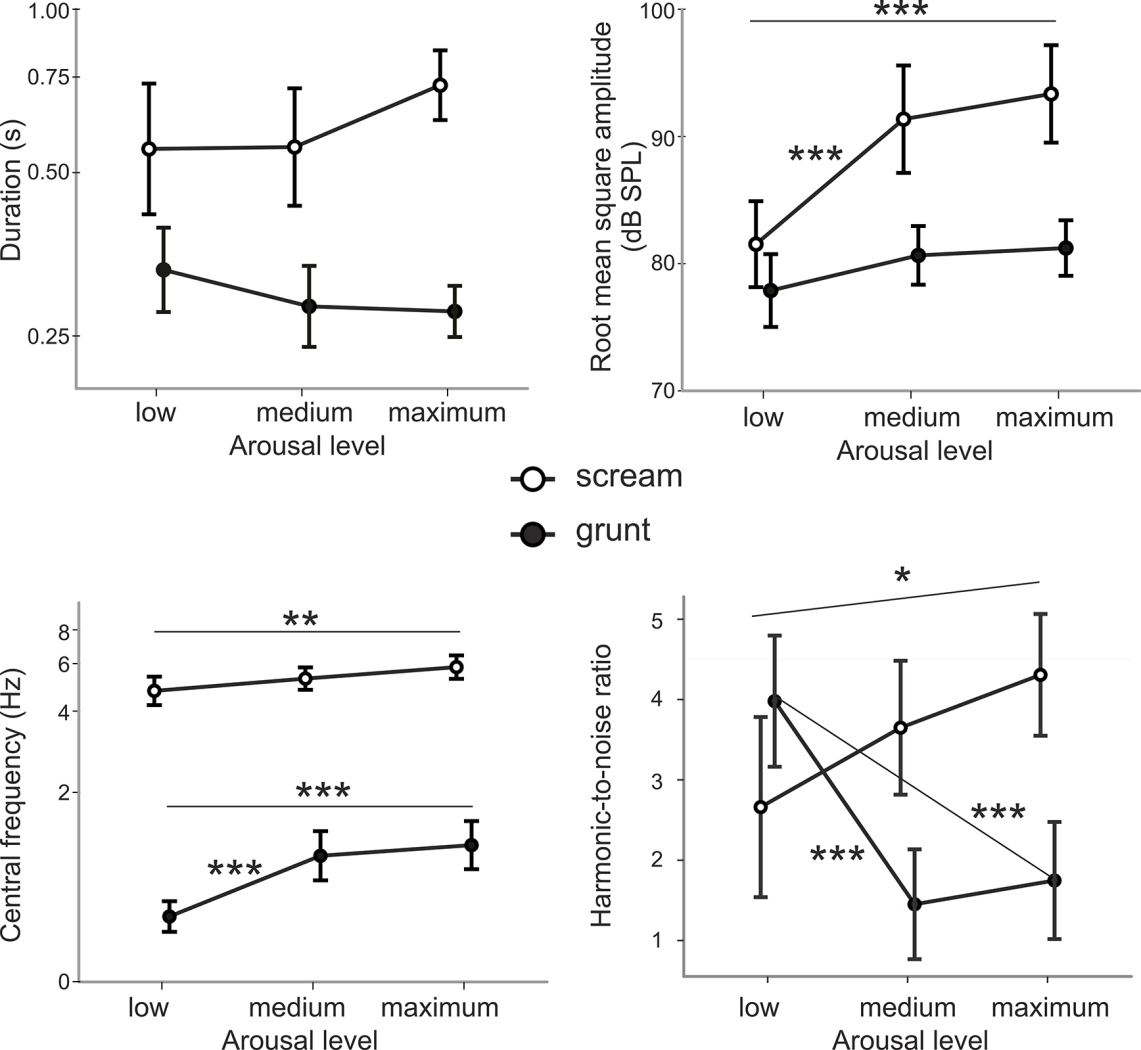
Scream (empty circles) and grunt (full circles) parameters in the three arousal levels. Means and 95% confidence intervals are shown. Duration and central frequency are displayed in logarithmic scale. N = 180 (6 calls from 30 individuals).

Screams differed from grunts in all of the investigated acoustic parameters (all p< 0.001, see the effect of calls type in Table 2 for details). The arousal level significantly affected the intensity and central frequency of the calls. With increasing arousal, calls became louder and higher. However, all of the acoustic parameters were also influenced by the interaction between call type and arousal level, suggesting that the two call types responded differently to changing arousal levels (see Fig 2), which was confirmed by post-hoc tests (S1 Table).

**Table 2:** Effects of call type (scream / grunt), arousal level (LOW / MEDIUM / MAXIMUM) and their interaction on the four acoustic parameters.

Variable	effect	df	F	p
Duration (log)	call type	1, 145	99.18	< 0.001
	arousal	2, 145	0.95	0.389
	call type * arousal	2, 145	3.29	0.040
Root-mean-square amplitude	call type	1, 145	100.00	< 0.001
	arousal	2, 145	28.18	< 0.001
	call type *arousal	2, 145	8.82	< 0.001
Central frequency (log)	call type	1, 145	772.67	< 0.001
	arousal	2, 145	13.27	< 0.001
	call type *arousal	2, 145	5.29	0.006
Harmonic-to-noise ratio	call type	1, 145	13.29	< 0.001
	arousal	2, 145	2.04	0.134
	call type *arousal	2, 145	15.48	< 0.001

The call duration did not significantly differ between the arousal levels for either of the two call types (S1 Table, all p > 0.19). The significant arousal *call type interaction thus probably arose from the slight, non-significant extension of the screams and shortening of the grunts with increasing arousal.

The intensity increased with arousal only in the scream calls (S1 Table, for grunt calls all p > 0.18); LOW screams were less intense than either MEDIUM (z = -6.43, p < 0.001) or MAXIMUM screams (z = -7.73, p < 0.001).

The central frequency increased in both the scream and grunt calls (S1 Table). In screams, the LOW arousal level differed only from the MAXIMUM arousal level (z = -3.64, p = 0.002), while in grunts the LOW arousal level differed from both the MEDIUM (z = -3.85, p < 0.001) and MAXIMUM arousal levels (z = -4.53, p < 0.001). The central frequency increased much more in the grunt calls than in the scream calls.

The harmonic-to-noise ratio increased with arousal in the scream calls (S1 Table). LOW arousal screams were less harmonic than MAXIMUM arousal screams (z = -3.02, p = 0.020). In contrast, the harmonicity of the grunts decreased with arousal, as both MEDIUM (z = 4.64, p < 0.001) and MAXIMUM (z = 4.10, p <0.001) grunts were less harmonic than the LOW arousal grunts.

## Discussion

In this study, we tested whether the acoustic structure of piglet distress calls and contact calls encoded cues to emotional arousal, and how specific acoustic features signal the level of arousal across the two call types. We found that piglets increase the grunt and scream rate when aroused (i.e. struggling during backtest). Increased calling rate has been often found to be associated with increased arousal [4]. Further, we found that the acoustic structure of both screams (distress calls) and grunts (contact calls) does vary with arousal. This is in agreement with previous studies of pig vocalizations [14,16] and, more generally, with theoretical and experimental studies showing how emotional state affects the physiology of an organism, which is in turn reflected in its vocalizations [1,7,20]. Such physiological changes are assumed to affect the production of all types of vocalizations, as documented by the wide range of contexts in which calls have been found to reflect emotional state [4,5]. Indeed, signalling situation urgency precedes the contextual specificity of calls [35].

Although the emotional state was signalled in both call types, we found that arousal was encoded differently in screams and grunts. The effect of situational arousal on the acoustic structure of two call types or more have previously been studied, but call parameters were averaged either from a subset of calls [36] or from all of the calls [14] within the same experiment without considering the actual physiological state in which the calls were emitted. Our study is the first to directly compare how emotional state is encoded in two different call types under the same conditions. The physiological conditions on the vocal folds (e.g. tension, subglottal pressure, etc.) and in the vocal tract (e.g. salivation) are likely the proximate factors affecting expression of emotions in vocalizations [4,8,20]. However, they are difficult to control and measure in live animals. The advantage of our study was that we analysed screams and grunts emitted in close succession (within 2 seconds) in each situation. The fact each scream and grunt pair was emitted in close succession allows us to confidently assume that the emotional and physiological state was comparable for both call types.

We found significant interactions between arousal level and call type for all of the four acoustic parameters. These results indicate that the function and/or structure of call types affects the acoustical encoding of emotions. Such effects could explain why studies investigating vocal correlates of emotions in animals vocalisations [4,5] as well as in human speech or vocalisations [37] have found different and sometimes contradicting results. Piglet grunts are contact calls with an acoustic structure suitable for short-range communication (e.g. short duration and low intensity), while screams are distress calls 'designed' to be heard (with e.g. a long duration and high intensity). We suggest that constraints linked to the production of these two call types [24] cause the observed differences in the encoding of emotions.

Neither the duration of the screams nor the duration of the grunts was significantly affected by arousal. Despite the fact that the relationship between call duration and arousal has received much scrutiny, the results have been contradictory: increasing arousal has been found to be associated with either an increase, a decrease, or no change in call duration [4,5]. Increased call duration helps detection and localization[38], and conflicting results are particularly reported in alarm and isolation contexts [5] when facilitating localization may not always be beneficial and might, for instance, attract unwanted attention from predators. Hence, the context of call emission and whether the call is designed to be easily detected or not could constrain the way that emotional arousal is expressed in call duration.

Both screams and grunts increased in central frequency with increasing arousal. This is in accordance with the general trend observed across mammalian species that call pitch increases with arousal [4,5]. Two aspects of vocal production are likely to affect the pitch of screams and grunts. First, heightened activity when the piglets were struggling to escape (MEDIUM and MAXIMUM arousal levels) is likely to result in increased muscle tension, breathing rate and breathing amplitude, leading to an increase in the subglottal pressure and higher F0, which can, in turn, influence the energy spectrum including the central frequency. Second, the need for an increased air supply during struggling may result in more frequent and greater mouth opening, thereby raising the formant frequencies [7,20]. We suggest that differences in the production of screams and grunts could explain why the rate of increase is higher for grunts. Loud calls such as screams are typically emitted orally with an open mouth [24]. Although grunts are typically emitted nasally with a closed mouth in states of low arousal [24], our piglets frequently opened the mouth whilst producing grunts in high levels of arousal, thus considerably shortening the length of the vocal tract and modifying its resonance properties[24].

High activity connected with intense breathing during struggling (MEDIUM and HIGH arousal levels), causes higher subglottal pressure [7,20], and should lead to an increase in the amplitude of both call types. Increasing call amplitude with arousal has been found across a wide range of species and contexts [4]. However, the amplitude increased significantly only for screams, while the amplitude of the grunts was not affected by arousal. This may be a consequence of the fact that grunts are relatively faint vocalizations used in close range communication, and that because grunts are typically produced nasally, this does not allow for a substantial increase in amplitude. Another contributing factor could be that nasal cavities absorb more acoustic energy than oral cavities [24]. More work on the mechanisms of vocal production in pigs is clearly needed to confirm or refute these speculations.

We also found that the harmonic-to-noise ratio showed opposite changes for screams and for grunts–it increased with arousal in screams while it decreased in grunts. Previous laboratory studies have described transitions to chaotic vibration regimes with increasing subglottal pressure [39,40]. However, this does not seem to happen with piglet screams. In our study, the harmonicity of the scream calls actually increased (i.e. chaotic vibrations decreased) with arousal, as previously observed in screams emitted in the extremely painful situation of unanesthetised castration [16]. Increased tonality in calls given in more urgent situations has also been documented in other mammal species [11]. It is possible that certain call types, including piglet screams, need substantial air flow before they attain optimum phonation. It has indeed been suggested that, in humans, increased jitter (= lower voice tonality) could be associated with both hypotension and hypertension of the vocal folds [20].

The acoustic structure changed more profoundly in the scream calls (affecting three out of four measured parameters). Scream calls can be viewed as distress calls because they are mostly emitted in contexts of immediate danger [22]. It is possible that the acoustic structure of distress calls is particularly adapted to either honestly reflect the state and need of the piglet [15,41] or to elicit an appropriate response from receivers [42]. The similar structure of infant distress calls observed in many species [43,44], conforming to the 'motivational-structural rules’ proposed by Morton and followers [18,45] may reflect these adaptation pressures.

In conclusion, our study documents that relationship between emotions and expression of emotions through vocalizations is rather complex. We suggest that besides taxa-specific patterns of emotional prosody [46], constraints linked to the specific structure and function of call types could explain some discrepant results reported on the acoustic expression of emotions. Our study provides additional evidence supporting the idea that emotions and associated physiological changes affect vocalization features. However, the way emotions are encoded depends on the specific type of vocalization, probably reflecting constraints exerted by its production and its function. Distinguishing the relative contribution of structure vs. function (though both may be inter-connected as well) to limiting or promoting emotional expression in vocalizations will require comparative studies of call types differing in function but with a similar structure and vice versa.

## Supporting Information

S1 File Dataset for the analysis of effects of call type (scream / grunt), arousal level (LOW / MEDIUM / MAXIMUM) and their interaction on the four acoustic parameters. (CSV)

S1 Table Results of posthoc tests for comparisons of screams and grunts in three levels of arousal (n = 30 individuals). (DOCX)
