# Supplementary material for: Expression of Emotional Arousal in Two Different Piglet Call Types
Source: PLoS One. 2015 Aug 14;10(8):e0135414. doi: 10.1371/journal.pone.0135414 (PMC4537126; doi:10.1371/journal.pone.0135414)
Supplement: S1 Table — (DOCX) [file pone.0135414.s002.docx]

# Supporting Information

**S1 Table.** Results of posthoc tests for comparisons of screams and grunts in three levels of arousal (n = 30 individuals).

|  |  | z | p |
| --- | --- | --- | --- |
| log10 duration | |  |  |
|  | scream LOW x scream MEDIUM | -0.06 | 1.000 |
|  | scream LOW x scream MAXIMUM | -2.17 | 0.192 |
|  | scream MEDIUM x scream MAXIMUM | -2.11 | 0.219 |
|  | grunt LOW x grunt MEDIUM | 1.24 | 0.757 |
|  | grunt LOW x grunt MAXIMUM | 1.42 | 0.644 |
|  | grunt MEDIUM x grunt MAXIMUM | 0.17 | 1.000 |
| amplitude | |  |  |
|  | scream LOW x scream MEDIUM | -6.43 | <0.001 |
|  | scream LOW x scream MAXIMUM | -7.73 | <0.001 |
|  | scream MEDIUM x scream MAXIMUM | -1.30 | 0.722 |
|  | grunt LOW x grunt MEDIUM | -1.81 | 0.379 |
|  | grunt LOW x grunt MAXIMUM | -2.19 | 0.185 |
|  | grunt MEDIUM x grunt MAXIMUM | -0.38 | 0.998 |
| log10 central frequency | |  |  |
|  | scream LOW x scream MEDIUM | -1.88 | 0.335 |
|  | scream LOW x scream MAXIMUM | -3.64 | 0.002 |
|  | scream MEDIUM x scream MAXIMUM | -1.76 | 0.408 |
|  | grunt LOW x grunt MEDIUM | -3.85 | <0.001 |
|  | grunt LOW x grunt MAXIMUM | -4.53 | <0.001 |
|  | grunt MEDIUM x grunt MAXIMUM | -0.68 | 0.977 |
| hnr | |  |  |
|  | scream LOW x scream MEDIUM | 2.02 | 0.247 |
|  | scream LOW x scream MAXIMUM | 3.30 | 0.007 |
|  | scream MEDIUM x scream MAXIMUM | 1.29 | 0.718 |
|  | grunt LOW x grunt MEDIUM | 1.78 | 0.381 |
|  | grunt LOW x grunt MAXIMUM | 1.23 | 0.757 |
|  | grunt MEDIUM x grunt MAXIMUM | -0.62 | 0.983 |

Only comparisons within call types are presented to show how particular call type changes with arousal.
